# Evaluating the effectiveness of undergraduate clinical education programs

**DOI:** 10.1080/10872981.2020.1757883

**Published:** 2020-04-30

**Authors:** John W. Ragsdale, Andrea Berry, Jennifer W. Gibson, Christiane R. Herber-Valdez, Lauren J. Germain, Deborah L. Engle, representing the Program Evaluation Special Interest Group of the Southern Group on Educational Affairs (SGEA) within the Association of American Medical Colleges (AAMC)

**Affiliations:** a Assistant Dean for Clinical Education, University of Kentucky College of Medicine, Lexington, KY, USA; b Executive Director of Faculty Life, University of Central Florida College of Medicine, Orlando, FL, USA; c Director, Office of Medical Education, Tulane University School of Medicine, New Orleans, LA, USA; d Assistant Professor, Department of Medical Education, Paul L. Foster School of Medicine, Texas Tech University Health Sciences Center at El Paso, El Paso, TX, USA; e Managing Director, Office of Institutional Research and Effectiveness, Texas Tech University Health Sciences Center at El Paso, El Paso, TX, USA; f Director of Evaluation, Assessment and Research; Assistant Professor, Public Health and Preventive Medicine, SUNY Upstate Medical University, Syracuse, NY, USA; g Assistant Dean, Assessment and Evaluation, Duke University School of Medicine, Durham, NC, USA

**Keywords:** Undergraduate medical education, clinical education, program evaluation, accreditation, curriculum

## Abstract

Medical schools should use a variety of measures to evaluate the effectiveness of their clinical curricula. Both outcome measures and process measures should be included, and these can be organized according to the four-level training evaluation model developed by Donald Kirkpatrick. Managing evaluation data requires the institution to employ deliberate strategies to monitor signals in real-time and aggregate data so that informed decisions can be made. Future steps in program evaluation includes increased emphasis on patient outcomes and multi-source feedback, as well as better integration of existing data sources.

## Introduction

Undergraduate medical education programs are charged with evaluating learner activities during clerkship experiences and the outcomes of their learning, in order to determine program effectiveness. Program evaluation should involve a three-pronged approach that includes baseline measurements (pre-clerkship), process measurements (activities of learners during the clerkship) and outcome measurements (assessment of learning products or end points)[1]. Many of these measures are defined by the Liaison Committee on Medical Education (LCME) in its various educational standards but are not aggregated in a succinct resource to facilitate this process. In addition, the LCME documents do not explicitly categorize these concepts in terms of the types of measures being used (e.g., process, outcome). We believe that by aggregating these standards and by applying existing educational frameworks, we can improve the effectiveness of program evaluation for the highly complex, clinical training environment.

LCME Element 1.1 requires medical schools to engage in strategic planning and continuous quality improvement (CQI) processes that establish short- and long-term programmatic goals, result in the achievement of measurable outcomes that are used to improve programmatic quality, and ensure effective monitoring of the medical education program’s compliance with accreditation standards [2]. A robust approach to program evaluation can help ensure sufficient attention is given to critical measures to avoid a ‘severe action decision’ by the LCME [3]. To meet these goals, we are presenting a summary of the data to be included in the program evaluation process and a discussion of strategies to be used in collecting and reviewing that data. We believe that having a comprehensive and succinct list of process and outcome measures will allow faculty and administrators to more effectively monitor, assess, and evaluate the quality of their educational programs as part of the CQI process. In this paper, we propose a set of guidelines or best practices that can be used by all parties responsible for program evaluation to identify essential data sources, as well as mechanisms to access, monitor, and analyze data to determine program effectiveness.

## Types of measures

Data to evaluate program effectiveness can broadly be grouped into process measures and outcome measures. Process measures focus on aspects of program and curriculum delivery, such as logistics of how teaching occurs, how courses are organized, and the types of patient encounters required in the curriculum. These measures may be granular (e.g., the number of duty hour violations in a clerkship) or more broad (e.g., how formative feedback is provided), but all evaluate interim steps or components in the learning process, not the result of the process. Outcome measures, in contrast, evaluate if learning occurred, particularly whether student- and program-level objectives and targets were met. These can also be granular (e.g., passing rate on standardized tests) or broad (e.g., successful transition to residency). These categories of process and outcome measures are not strictly defined, but are important constructs to consider in the design of evaluation strategies and the selection of measures for determining program effectiveness. By viewing program evaluation through this lens of process and outcome measures, stakeholders can ensure they are considering program evaluation broadly and can better prioritize different types of measures. Since outcome measures focus on the end products of learning, these measures should be weighed more heavily than process measures, though both are important.

Another model that is useful to consider is the Kirkpatrick model of evaluation [4–8]. First proposed by Donald Kirkpatrick in the 1950s [9],this model includes four levels of outcomes for a training program: reaction, learning, behavior, and results. Since then, the framework has been expanded and revised during its extensive use. Its most recent iteration, the New World Kirkpatrick Model [4], expands on the original four-level model based on the effect on learning outcomes:

- Level 1: Reaction

This includes learner satisfaction, engagement, and relevance.

- Level 2: Learning

This includes changes in knowledge, skills, attitudes, confidence, and commitment.

- Level 3: Behavior

This includes the application of what was learned and change in learner behavior.

- Level 4: Results

This includes the achievement of outcomes and indicators of progress towards those outcomes.

This model can be very useful in evaluating clinical program effectiveness. For example, course evaluation ratings would be considered Kirkpatrick level 1 and measures of medical knowledge on standardized tests would be Kirkpatrick level 2. Applying the Kirkpatrick framework to measures of program effectiveness can help stakeholders prioritize different measures. For example, measures of behavior change are more meaningful than measures which simply reflect learner satisfaction, though the former may be harder to demonstrate. In addition, this framework can indicate opportunities to improve program evaluation rigor by highlighting measures to include at higher Kirkpatrick levels.

Applying both of the above frameworks, we compiled a summary of essential measures to use in evaluating the effectiveness of undergraduate clinical education programs (Table 1.).

**Table 1.** Essential measures to use in evaluating the effectiveness of undergraduate clinical education programs

Categories		Specific Measures	Corresponding LCME Substandard(s)	Kirkpatrick Level[^4,5]^
Outcome Measures	Performance on national assessments	USMLE Step 2 CK	8.4	2
		USMLE Step 2 CS	8.4	2, 3
		NBME Clinical Subject Examinations	9.0	2
	Performance on local assessments	Local medical knowledge assessments	8.4, 9.0, 9.4, 9.8	2
		Objective Structured Clinical Examinations (OSCEs)	8.4, 9.0, 9.4	2, 3
		Clinical evaluations	9.0, 9.4	2, 3
		Other measures of competency attainment or entrustment (e.g., mini-CEX)	6.1, 9.0, 9.4	2, 3
	National measures of student satisfaction	AAMC Graduation Questionnaire	Throughout	1, 2
	Local measures of student satisfaction	Clerkship/course evaluations	8.3, 8.5	1, 2
		Faculty teaching evaluations	8.3, 8.5	1, 2
		Resident teaching evaluations	8.5	1, 2
	Career outcomes	Specialty selection	8.4	1, 2, 3
		Residency matching results	8.4	2
		Performance in the first year of residency (self and program director evaluations)	8.4	3
Process Measures	Curriculum design	Clerkship and session learning objectives	6.1, 8.3	1, 2
		Instructional formats and quality of instruction	6.0, 7.2, 8.3	1
		Assessments aligned to learning objectives	8.3, 9.0	1, 2
		Grade distribution	9.0, 9.6, 9.8	2
		Formative feedback, including mid-clerkship feedback	9.0, 9.7	2, 3
		Direct observation	9.4	2, 3
	Clinical experiences	Clinical settings/sites	5.5, 6.4	1
		Clinical roles and expectations	3.5, 6.1	1
		Required clinical experiences	6.2, 8.6	1, 2
		Patient volumes	5.5	1
	Learning environment	Physical spaces and resources	5.4, 5.6, 5.8, 5.9, 5.11	1, 4
		Supervision	9.3	1, 4
		Security and safety	5.7	1, 4
		Mistreatment	3.6	1, 4
	Preparation of instructors	Faculty	4.5, 6.1	1, 2, 4
		Residents	6.1, 9.1	1, 2, 4
	Compliance	Grade submission	9.8	-
		Duty hours	8.8	-
		Other university/college policies	4.6	-
Comparability across sites/campuses is relevant to most of the above measures (if applicable)			8.7	1, 2, 3

## Strategies for tracking and monitoring data

Program evaluation data are collected both within medical schools and by at least four external regulating bodies: the Liaison Committee on Medical Education (LCME), National Board of Medical Examiners (NBME), Association of American Medical Colleges (AAMC), and National Resident Matching Program (NRMP). These data are critical in decision-making at the local level (e.g., improvement in individual clerkships) and national level (e.g., LCME accreditation). However, using data effectively for decision-making requires aggregating them across different sources, developing internal processes to ensure data integrity, and enacting a deliberate strategy for data management. An inventory of data sources currently in use can be a valuable first step for organizing the process and gathering stakeholder input. Such an inventory can be structured by: a) level of data (e.g., individual student, clerkship group, graduation cohort, exam, clerkship, year, program), b) data source, c) party responsible for the review, d) data storage location, e) output/report format, f) collection/reporting cycle, and g) reviewers/data users. During the inventory process, it is important to develop protocols for managing data, including business process rules for data input and flow across systems, data definitions, and limitations. For example, annual NBME exam performance reports include data for each institution’s entire group of test takers, which might not correspond to academic year cohorts due to misalignment with the institution’s academic calendar or students delaying the exam. In addition, de-identification of data and procedures for the dissemination and sharing of data sets are necessary to safeguard student records. When the inventory is complete, data collected at the same level can be organized by identifiers such as student ID or clerkship name, and merged manually or automatically aggregated. The use of an education data warehouse may facilitate this process [10]. Maintaining data architecture, hygiene, and quality assurance processes are critical to success.

The use of data visualization tools, such as online dashboards, allows for customizable summaries and real-time reporting, while making data more accessible and interpretable for stakeholders. AAMC’s Curriculum Dashboard Resource [11] lists four primary reasons to develop curriculum dashboards: ‘compare metrics to national standards, evaluate educational programs over time, identify trends in educational program quality, and benchmark faculty, resident and student performance.’ Stonybrook’s Drivers of Dashboard Development (3-D) approach [12] is used in curricular CQI and has been linked to improvements in LCME compliance activities, including timeliness of grades, mid-clerkship feedback, and policy awareness. The most critical elements to consider in dashboard creation are who the end-users are, their level of data fluency, and how the data will be used in decision-making. It is also important to undergo a standard-setting process to determine appropriate benchmarks for each metric.

Data-driven decision-making regarding clinical education programs occurs on a variety of cycles. While some metrics can be reviewed annually, others require immediate or near immediate action. An incident of mistreatment reported on an end-of-clerkship evaluation, for example, necessitates a rapid response, which can be activated by an automatic alert informing the responsible parties of the issue. Data alerts are important but should be used sparingly, to avoid unnecessarily fatiguing those responsible for responding. Queries of stakeholders, existing policies, and accreditation expectations will determine in which circumstances and at which time points alerts are necessary. Often alerts are associated with sensitive information like poor performance and problems with the learning environment, and therefore, a consistent response procedure should exist and be made transparent to students, faculty, staff, and other stakeholders. Alert response procedures should include to whom the alerts will be sent, the type of information they will include (particularly if identifying data are involved), and action steps to be taken.

## Strategies for using data for curriculum oversight

Data play an important role in determining the quality of the educational program and whether the program meets the goals and expectations of its stakeholders. This process can be used to inform the future direction of the curriculum and essential functions that support the curriculum, such as faculty development. Most LCME-accredited programs utilize standardized data provided by national sources such as the NBME and AAMC, as well as internal information. Information provided by external sources allows a program to benchmark outcomes against national percentiles. Internal sources of information can be useful in detecting and evaluating contextual features unique to a program.

Informed by LCME requirements, ‘medical schools must collect and use a variety of outcome data, including national norms of accomplishment, to demonstrate the extent which medical students are achieving program objectives and to enhance the quality of the medical education program as a whole.’[13] Under the oversight of the institution’s curriculum committee, valid data must be collected to ensure the trustworthiness of information and to eliminate anecdotal storytelling, which can undermine the curriculum[14]. Additionally, as the final authority on curricular matters, the curriculum committee has to review relevant data in order to make curricular decisions and improvements. Many schools determine cut-off measures – often through a curricular dashboard – to highlight strengths which can then be replicated in other areas of the curriculum, or weaknesses that require additional resources, support, or monitoring. Outcomes of such a review can also be used to inform faculty development to address areas of weakness in the curriculum.

## Future steps in program evaluation

Typically, the evaluation of clinical programs relies on a combination of learner satisfaction (Kirkpatrick Level 1 [4]), measures of learning (Kirkpatrick Level 2), and changes in behavior (Kirkpatrick Level 3). However, the latter is limited by a paucity of nationally standardized measurement tools. The goal of a clinical education program is to graduate clinicians who can function effectively in their professional roles and provide high-quality care. To determine if this goal is being achieved requires measuring the care that is delivered by the program’s graduates, that is, by measuring the effects of the clinical program on patients (Kirkpatrick Level 4).

With a rapidly changing healthcare landscape and increasing public demands for accountability, the discourse on evaluation frameworks are shifting towards measures of patient outcomes [15,16]. As the Institute of Medicine [17] highlighted the need for clinical education to fit healthcare needs, calls to examine the effects of educational training on the quality of care provided by health profession learners followed [15,16,18–22]. Early responses included recommendations for ‘evidence-guided education’, whereby medical educators monitor clinical outcomes to inform the design of medical education programs [23]; others called for the development of research agendas to examine the impact of educational programs on clinical outcomes[18]. Though methodological challenges and factors that confound the performance of medical professionals have been acknowledged [16,18,20,24], there has been a general consensus on the need to include population outcome measures in the evaluation of clinical teaching strategies, curricula, and programs. Recently, patient-reported outcomes (PROs) and patient-based outcomes (PBOs) have been discussed as critical indicators for program evaluation and continuous quality improvement [15,16]. While some studies have examined clinical outcomes as measures of education quality [25–30], uniform systems and efficient ways of collecting and analyzing outcome data across institutions are needed [15,22]. Ultimately, the primary goal of clinical education is to prepare professionals who deliver quality healthcare; hence, the goal of evaluation should be to demonstrate that clinical education programs are contributing to improved patient outcomes.

In evaluating program effectiveness, it is important to include a variety of perspectives. For example, assessment of student performance during clerkships should not only include evaluations from faculty and residents, but also from patients, clinical staff, administrative staff, peer students, and even self-evaluations. Multisource Feedback (MSF) approaches, such as 360-degree evaluations, are already used in many residency training programs [31–36] and even in some undergraduate medical education programs [37,38]. MSF evaluations can provide valuable insight into the learning environment, increase stakeholder representation in the medical education program, and identify gaps in skill development that may go unrecognized in traditional evaluations.

Beyond additional types of data, future steps in program evaluation also include better data systems and more robust data-tracking mechanisms. Currently, most program measures exist in systems that do not communicate well with one another. This makes integration into a coherent database that provides real-time updates challenging. For example, Graduation Questionnaire data are only initially provided in Portable Document Format (PDF) format rather than a format which allows for integration into a data management system. A future state in which raw data, especially nationally normed data, is provided electronically in formats which integrate with other local data systems would allow for better tracking of program data and assessment of interventions in real-time.

## Conclusion

As stakeholders evaluate the effectiveness of clinical education programs, it is important to understand the types of measures that must be included and how these measures relate to each other. It is also imperative that programs have a robust mechanism to track and monitor data and use it to inform curricular decisions. As types of data and data systems evolve, we will be better able to accomplish these goals and ensure our clinical education programs are effective in training future providers.
